# Identification of Pre- and Post-Treatment Markers, Clinical, and Laboratory Parameters Associated with Outcome in Renal Cancer Patients Treated with MVA-5T4

**DOI:** 10.3389/fonc.2013.00185

**Published:** 2013-07-15

**Authors:** Rabih Said, Robert J. Amato

**Affiliations:** ^1^Division of Oncology, Department of Internal Medicine, Memorial Hermann Cancer Center, University of Texas Health Science Center at Houston (Medical School), Houston, TX, USA

**Keywords:** cancer immunotherapy, TroVax, biomarkers

## Abstract

The recent approvals of immunotherapeutic agents (Sipuleucel-T and Ipilimumab) for the treatment of different solid tumors gave a boost to the growing cancer immunotherapy field, even though few immunotherapy studies have demonstrated convincingly that there is a direct link between the predicted mode of action of an immunological compound and therapeutic benefit. MVA-5T4 (TroVax^®^) is a novel vaccine combining the tumor-associated antigen 5T4 to an engineered vector-modified vaccinia Ankara (MVA). MVA helps to express the oncofetal 5T4 antigen and subsequently trigger a tumor-directed immune reaction. The safety and clinical benefit reported in multiple phase I and II clinical trials using MVA-5T4 were encouraging; immune responses were induced in almost all treated patients, and associations between 5T4-specific cellular or humoral responses and clinical benefit were reported in most of the nine phase II trials. In particular, clinical studies conducted in renal cell carcinoma (RCC) patients have demonstrated an association between 5T4-specific (but not MVA) antibody responses and enhanced survival. This review describes the clinical studies using MVA-5T4 conducted in RCC that convincingly demonstrated that an antigen-specific immune response induced by vaccination is associated with enhanced patient survival and is not simply a function of the general “health” of patients. We will also provide our expert opinions on possible future better-designed clinical trials based on relevant biomarkers. In addition, various combinations of MVA-5T4 and different and newer immunomodulator agents with promising clinical benefit will be discussed.

## Introduction

The incidence of renal cell carcinoma (RCC) varies widely from region to region, with the highest rates observed in North America and Czech Republic (1). It is the tenth most common cancer in Europe (2). In the United States, there are approximately 65,000 new cases and almost 14,000 deaths from RCC each year (3). RCC includes several different histologic subtypes that possess distinct biological behaviors and prognoses. Approximately 75% of all RCCs are of the clear cell type, which is associated with the worst prognosis (4). Advanced RCC is highly resistant to chemotherapy, and cytotoxic drugs generally only have a small effect on tumors in a very selective subgroup (5, 6). In a small percentage of patients, high-dose interleukin-2 (IL-2) can be very effective, but it is poorly tolerated (7). Recent research has focused on developing molecular-targeted therapies (e.g., sunitinib, sorafenib, bevacizumab, axitinib, temsirolimus, and pazopanib), many of which have been approved for the treatment of metastatic RCC. The role of vaccine therapy in the management of various solid tumors, including RCC, is rapidly evolving; HSPPC-96 (Vitespen^®^), protein peptide complex consisting of a 96-kDa heat-shock protein, was tested in a phase III clinical trial and showed some clinical benefit in terms of increased recurrence-free survival in patients with intermediate risk (8); in fact, this agent is currently approved in Russia for treatment of RCC (9). IMA-901, a novel vaccine consisting of 10 synthetic tumor-associated peptides, is currently being studied in combination with either sunitinib, cyclophosphamide (CY), or granulocyte macrophage-colony stimulating factor (GM-CSF) in a phase III trial (NCT01265901).

Currently, the standard of care (SOC) for non-metastasized RCC includes cytoreductive surgery without any adjuvant treatment outside clinical trial setting, while the SOC for metastasized RCC include cytoreductive surgery, if feasible, followed by any of the various molecular-targeted therapies (10). In addition to the anatomical extent of the disease, other poor prognosis factors, including a low Karnofsky performance status (<80%), high serum lactate dehydrogenase (>1.5 times upper limit of normal), low hemoglobin (<lower limit of normal), high “corrected” serum calcium (>10 mg/dL), and absence of prior nephrectomy, have been validated as predicting shorter overall survival (11, 12).

Although newer targeted agents improve the overall outcome of patients with RCC, the median survival of patients with advanced disease is still relatively poor (13). Therefore, new agents for the treatment of RCC are needed. Vaccine therapy is a growing class of drugs for the treatment of various cancers, including RCC. The development of cancer vaccines has also been explored using viral vectors to promote immune responses against tumor-specific antigens (14, 15). Viral vector-based vaccine therapy involves identifying (i) tumor-associated antigens (TAAs) and (ii) the most suitable delivery system. Cancer cells generally show some degree of genetic instability (16), which can lead to the production of an assortment of aberrant proteins that are hypo- or hyper-glycosylated as well as highly expressed or expressed during the wrong development stage (17). An optimal TAA is minimally expressed (if at all) in normal tissue yet still has homogenous high-level expression on the surface of a broad range of tumors. In particular, TAAs that are associated with disease progression are optimal because they actively interfere with tumorigenesis in addition to attacking the tumor. Once a TAA is selected, the method of delivering it to the immune system must be considered. Proteins expressed by carcinomas are usually presented to the immune system as self-proteins, which elicit no immune response (18). By contrast, using a viral delivery system breaks self-tolerance and elicits an immune response to the TAA.

One of the most commonly used viral delivery system is modified vaccinia Ankara (MVA) because of its well-documented safety profile and proven ability to generate potent immune responses (19, 20). A promising TAA is the 5T4 antigen, which is normally expressed in the placenta and rarely detected on most normal adult tissue (21, 22), although it is highly expressed in a range of human carcinomas (23–25).

## The Vaccine

MVA-5T4 (TroVax^®^) is a novel vaccine, using MVA as a vector, which was engineered to induce the expression of the oncofetal 5T4 TAA on tumor cells. Subsequently, the vaccine can break the immune tumor tolerance through the induction of cellular and humoral responses to both MVA and 5T4; therefore, it provides protection against 5T4-positive tumors (26, 27).

Tumor-associated antigens belong to one of three major categories: (i) non-self-viral antigens (e.g., E6/E7 from human papilloma virus), (ii) altered self-antigens (e.g., MUC-1), and (iii) non-mutated self-antigens (e.g., carcinoembryonic antigen). Human 5T4 belongs to the non-mutated self-antigens category. The 5T4 antigen is a 72-kDa transmembrane glycoprotein with an extracellular part composed of leucine-rich repeat domains separated by hydrophilic amino acids with 7 N-linked glycosylation sites (28, 29). The 5T4 antigen is normally highly expressed in the placenta but is also highly expressed in>80% of breast, renal, colorectal, prostate, and ovarian carcinomas (22, 30). Although the precise function of 5T4 in tumorigenesis has yet to be elucidated, *in vitro* human and murine studies have found that 5T4 is concentrated at the microvillus projections of the plasma membrane and that the overexpression of 5T4 alters cell adhesion, motility, and morphology (31). Overall, studies have shown that tumors expressing 5T4 antigen are associated with poor clinical outcome (23, 32–35).

Modified vaccinia Ankara is a highly attenuated strain of vaccinia virus – a member of the poxvirus family – that is unable to replicate in most mammalian cells (36). MVA was derived as a safe smallpox vaccine that showed no serious side effects; infected mammalian cells efficiently produce products of both early and late viral genes, as the block in viral replication occurs during virion assembly (37). In addition, its large double-stranded DNA genome is likely to accommodate>25 kb of foreign DNA (38).

MVA-5T4 has shown therapeutic effectiveness in preclinical models (39) as well as in clinical trials of patients with various solid tumors, including colorectal and prostate cancer (40–44). Herein, we will review all the clinical trials conducted in patients with metastatic RCC (mRCC; see Table 1). We will focus on both cellular and humoral immunologic responses and their correlation to the tumor response. We also will provide expert opinions for the better design of future clinical trials.

**Table 1:** **Summary of all clinical trials conducted using MVA-5T4 in the treatment of mRCC**.

	Study design	Treatment	Number of patients	Number of injections	MVA antibody response, *no. (%)	5T4-antibody response,*no. (%)	MVA ELISPOT, *no. (%)	5T4 ELISPOT, *no. (%)	Clinical benefit (RECIST)
Hawkins et al. (45)	Phase I/II	MVA-5T4 + INF-α	11	11	11 (100)	11 (100)	11 (100)	5 (45)	None
Amato et al. (48)	Phase II	MVA-5T4 + INF-α vs. MVA-5T4	28	9	25 (100)	21 (84)	14 (66)	7 (33)	PR: 1, SD^¤^: 5
Amato et al. (46)	Phase II	MVA-5T4 + IL-2	25	8	25 (100)	21 (84)	6 (54)	5 (45)	CR: 2, PR: 1, SD^¤^: 6
Kaufman et al. (47)	Phase II	MVA-5T4 + IL-2	25	8	23 (100)	23 (100)	23 (100)	13 (57)	CR^§^: 3
Amato et al. (49)	Phase III	MVA-5T4 + IL-2, INF-α or sunitinib	733	13	350 (96)	204 (56)	–	–	CR: 2, PR: 47, SD^¤^: 173

*Not all patients enrolled were tested. ^§^One patient had two abdominal masses that were removed surgically after regression, and two patients had complete regression of all metastatic sites after vaccine therapy and later underwent nephrectomy of the primary tumors. ^¤^Patients had SD for ≥6 months. CR, complete response; PR, partial response; SD, stable disease.

## Clinical Trials

### Phase I/II studies

An open-label phase I/II study was conducted in 11 mRCC patients who were receiving first-line systemic therapy (45). Patients with RCC received MVA-5T4 alongside interferon (IFN)-α. The primary objectives were to evaluate the safety, immunogenicity, and efficacy of the combination therapy. The treatment was well tolerated, and no significant adverse events were attributed to the treatment. The only MVA-5T4-related adverse event was soreness at the injection site. All patients mounted either boosted or *de novo* MVA-specific and 5T4-specific antibody responses as monitored by ELISA. The peak 5T4 and MVA antibody titers were achieved after 3–4 TroVax injections and remained constant thereafter. The IFN-γ enzyme-linked immunosorbent spot (ELISPOT) response was used to monitor antigen-specific cellular responses. There was no evidence that patients with preexisting MVA antibody had diminished 5T4-specific antibody response. All patients mounted MVA-specific ELISPOT responses, and six patients (54.5%) showed a greater than twofold increase in comparison to baseline. Out of 11 patients, 5 (45%) developed a 5T4-specific response. The median time to progression was 9 months (range, 2.1–18 months). No objective tumor responses were observed.

Three phase II clinical trials were conducted using MVA-5T4 in combination with IFN-α (46) and with IL-2 (46, 47). In a phase II clinical trial using MVA-5T4 with (*n* = 15) or without IFN-α (*n* = 13), safety, the immunologic response, and clinical efficacy were tested (48). As in the study described earlier, MVA-5T4 was found to be safe, without any serious adverse events or dose reduction. Immunological responses (cellular and humoral) of 23 patients showed that 91% (*n* = 21) and 100% (*n* = 23) of patients mounted boosted and *de novo* 5T4-specific and MVA-specific antibody response, respectively. Patients with preexisting MVA-specific antibody (*n* = 13) did not have a diminished 5T4-specific response. The IFN-γ ELISPOT assay performed on 21 patients showed that 14 of them had a ≥twofold increase (positive) in MVA-specific response. Out of 21 patients, 7 (33%) mounted a 5T4-specific ELISPOT response, including 1 patient with a weak (<fourfold increase) response. More patients mounted a specific-5T4 response in the MVA-5T4 group than in the combined group, but this was not statistically significant (38 vs. 25%). With respect to response to treatment, one patient in the combination group had a partial response (PR) lasting 7 months. No objective response was seen in the MVA-5T4-alone group. Overall, 14 patients (7 in each arm) had stable disease (SD), of whom 5 had SD for>6 months. The median PFS was 3.73 vs. 6.07 months (*p* = 0.13) and the median OS was 5.93 vs. 18.27 months (*p* = 0.02) for the group receiving MVA-5T4 with IFN-α vs. MVA-5T4 alone.

In addition, the median PFS was 10.2 months for patients who mounted a positive 5T4-specific ELISPOT response vs. 4.5 months for patients who did not (*p* = 0.04). OS was also increased in patients who mounted a positive 5T4-specific ELISPOT response, but this was not statistically significant (*p* = 0.21). Further analysis showed that patients with increases in 5T4 antibodies at week 7 (after the third vaccination) above the median fold showed a trend toward increased PFS and a significant improvement in OS (*p* = 0.01) compared to patients with antibody levels below the median. Correlations between outcome and MVA-specific antibody response using the same analysis were negative.

In a phase II open-label study, Amato et al. tested the safety, immunologic response, and clinical efficacy of MVA-5T4 vaccine in combination with IL-2 in mRCC (46). Twenty-five patients were enrolled in the study. MVA-5T4 was found to be safe with no serious adverse events or dose reductions. Out of 25 patients, 21 (84%) developed an antibody to 5T4, and all patients (100%) mounted MVA-specific antibody responses. The peak of both anti-5T4 and -MVA antibodies was achieved after three vaccine injections and remained relatively stable thereafter. Out of 11 patients, 6 (54%) and 5 (45%) showed positive MVA- and 5T4-specific ELISPOT responses, respectively.

Nine patients experienced a clinical benefit, including three patients (12%) with objective tumor responses (two CR and one PR) and six patients (24%) with SD for>6 months. Patients with objective responses mounted 5T4-specific antibody titers after three vaccinations that were higher than the overall median titer. The median PFS was 3.37 months (range, 1.5–24.76 months), and the median OS was 12.87 months (range, 1.90–24.76 months). The correlation between clinical and immunological responses showed significant differences in PFS among patients whose 5T4-antibody response was above the median compared with those whose 5T4-antibody response was below median (quantified between weeks 2 and 18; *p* = 0.015), but this was not true for the MVA antibody response. The OS data was immature, but a significant difference in OS was observed in patients whose 5T4-antibody response was above the median compared with those whose 5T4-antibody response was below the median between weeks 2 and 5 (*p* = 0.04); again, this was not the case for the MVA antibody response. The authors concluded that the combination of MVA-5T4 and IL-2 was well tolerated and safe, and the combination was planned for study in a phase III trial.

Another trial was conducted to test the efficacy of MVA-5T4 vaccination with IL-2 in 25 patients with mRCC (47). Patients were given the vaccine initially and then received a booster with IL-2 3 weeks later. Out of 25 patients, one patient withdrew from the trial (due to relocation), and another patient could not tolerate IL-2. The MVA-5T4 was well tolerated with no serious adverse events. All patients mounted boosted or *de novo* MVA- and 5T4-specific antibody responses as measured by ELISA. The IFN-γ ELISPOT assay showed a positive 5T4-specific response in 13 patients (57%) (SD, *n* = 10 vs. PD, *n* = 3) and an MVA-specific response in all patients.

Four-color flow cytometry showed that patients with SD had greater increase in CD8+CD107a+ T-cells than those with PD (1.50 ± 0.72 vs. 2.09 ± 0.30%, *p* = 0.015). Patients with PD showed significantly higher levels of PD-1 expressing CD4+ (*p* = 0.0329) and CD8+ T-cells (*p* = 0.0373) than patients with SD at 3 weeks. The absolute number of regulatory T-cells (T_REGs_) (CD4+CD25+FoxP3+) was decreased by 50% in patients with SD (*p* = 0.006). The effector/regulatory T-cell ratio was decreased in patients with PD but was both dramatically increased and maintained for up to 24 months in patients with SD.

The median PFS was 4.76 months, and the median OS was 28 months for patients with PD but had not yet reached for patients with SD at a median follow-up of 20 months (*n* = 12; *p* = 0.0206). The authors concluded that MVA-5T4 vaccine plus IL-2 can be safely given to patients with mRCC. Patients with SD were associated with increased levels of effector T-cells and decreased levels of T_REGs_ cells, which is suggestive a benefit from therapy.

### Phase III studies

To date, one phase III trial has been completed for MVA-5T4 in RCC patients (49). This randomized, double-blind, placebo-controlled study included 733 patients (365 MVA-5T4, 368 placebo) with mRCC. The primary endpoint was OS, and the secondary endpoints were PFS, objective response rate, and safety. Patients received up to 13 injections of MVA-5T4 or placebo in combination with one of the following: low-dose IL-2, IFN-α, or sunitinib. The study was terminated at the recommendation of the safety monitoring board because there was little or no prospect of demonstrating a significant survival benefit; however, patient follow-up continued. The median time on the study was 6 months, and only 5% of patients received a complete course of injections (*n* = 13). For patients who received IL-2 or IFN-α, the median number of MVA-5T4/placebo injections was eight, and for patients who received sunitinib, the median number of MVA-5T4/placebo injections was seven. The median OS was 20.1 and 19.2 months for the MVA-5T4 and placebo treatment arms, respectively. Patients with a good prognosis according to Memorial Sloan-Kettering Cancer Center (MSKCC) guidelines and who were treated with MVA-5T4 plus IL-2 showed significantly improved survival over those with a good prognosis who were treated with the placebo plus IL-2. The effect on OS of clinical baseline features and known prognostic risk factors was analyzed using a Cox proportional hazards model. It was noted that normal baseline levels of platelets (≤400 × 10^9^ cells/L), monocytes (≤0.80 × 10^9^ cells/L), and hemoglobin (age- and sex-specific ranges) seemed to affect the efficacy of MVA-5T4. Antibody responses against 5T4 and MVA were quantified, and patients who had a high 5T4-antibody response had improved survival over placebo-treated patients. In comparison, patients who had a high MVA-specific antibody response had no survival benefit compared to placebo-treated patients. Although the study results demonstrated that MVA-5T4 did not prolong survival when added to SOC therapies, this study revealed immune response surrogates that predicted longer survival, including normal pretreatment levels of platelets, monocytes, and hemoglobin. In addition, this phase III trial confirmed previous data reported in phase II trials that showed an association between 5T4 (but not MVA) antibody development and improved survival.

The data on numerous pretreatment factors associated with inflammatory anemia from this large phase III trial were later reanalyzed using a statistical modeling and showed that mean corpuscular hemoglobin concentration (MCHC) was the best predictor of a treatment benefit from MVA-5T4 (*p* < 0.01); this value was also positively correlated with tumor shrinkage (*p* < 0.001) and positively associated with 5T4-antibody response (50). These data were validated retrospectively using the data from phase II trials of MVA-5T4 in patients with colorectal, renal, and prostate cancers. In addition, tumor burden was found to be negatively correlated with MVA-5T4 treatment (50).

## Discussion

MVA-5T4 is a novel vaccine therapy that was engineered to express the 5T4 tumor-associated antigen, to break the immune tolerance toward tumor cells, and ultimately to treat various solid tumors. Preclinical data have shown encouraging results. Furthermore, early-phase I/II trials confirmed its safety profile with minimal grade 1–2 side effects. In addition to its safety profile, clinical effectiveness was noted in RCC, prostate cancer, and colorectal cancer. Early-phase clinical trials also showed a preliminary positive correlation between immune response to 5T4 and clinical outcome. Currently, this vaccine is being studied in mesothelioma (NCT01569919) and ovarian carcinoma (NCT01556841).

Unfortunately, the positive results seen in phase I/II clinical trials were not replicated and validated in the phase III clinical trial when MVA-5T4 was added to the SOC. Even though the phase III trial did not meet its endpoints, several points in the trial’s design need to be highlighted that might explain the less-than-expected results: (a) minimal imbalances in prognostic factors were not in favor of the MVA-5T4 arm; (b) the choice of the SOC was based on the local best practices; (c) the futility stopping rule was termination of MVA-5T4 treatment after a median time on study of only 6 months, which would affect the overall outcome given the fact that immunotherapy, unlike chemotherapy, has a delayed response (51); (d) the dosing and boosting schedule of MVA-5T4 might not have been optimal and a more accelerated schedule could be necessary in light of the fact that the T-cell responses after two or three vaccinations could be transient, as was seen in some of the earlier studies (40, 41); and (e) the role of predictive biomarkers to select patients for MVA-5T4 treatment is critical, especially given that the group with good MSKCC prognostic scores did very well with the IL-2+MVA-5T4 treatment.

In addition to the MSKCC prognostic score, further analyses of the data from all these clinical trials have shown that biomarkers including normal levels of platelet counts, hemoglobin and monocytes, and MCHC might predict a better outcome with MVA-5T4 treatment. Furthermore, the expression of 5T4 on tumor cells as a predictive biomarker for response to MVA-5T4 needs to be evaluated and studied. Determining the expression of 5T4 requires tumor tissue, which might be difficult in some settings; testing 5T4 on circulating tumor cells needs to be explored as an option that does not require tissue banking. All of these biomarkers should be considered in any future clinical trial design using MVA-5T4.

In addition to including the role of predictive biomarkers in future clinical trials design, the combination of MVA-5T4 with other immunomodulator agents in the treatment of RCC (and probably other solid tumors) will be of great interest and at least theoretically might add clinical benefit. Herein, we will propose some of the combinations that we think it is worthwhile to pursue.

The combination of MVA-5T4 with GM-CSF was previously studied in hormone-refractory prostate cancer, and it showed a high frequency of 5T4-specific immune responses that were correlated with longer time to progression (44). GM-CSF has demonstrated clinical benefit in the treatment of cancer patients on the basis of their immunomodulatory properties (enhancement of monocyte-mediated and dendritic cell-mediated antibody-dependent cellular cytotoxicity and T-cell cross priming) (52–54). This combination should be studied in mRCC, in which apoptosis of T-cells has been previously reported as an immune escape mechanism (55, 56).

T_REGs_ account for 5–10% of CD4+ T-cells, and they constitutively express CD25 (57). T_REGs_ control the key aspects of immune tolerance and play an important role in the lack of antitumor immune responses (58). CY is a chemotherapeutic agent with a dual effect on immune system: low-dose CY decreases the cell number and functionality of T_REGs_, which may enhance its antitumor activity (59). Therefore, the combination of low-dose CY and MVA-5T4 is a potentially successful strategy to overcome the immune tolerance of mRCC by enhancing the killing effect of the immune system at least at two different levels.

Treatment of mRCC has improved greatly over the past decade with the approval of multiple molecular-targeted therapies, including sunitinib, sorafenib, and temsirolimus, among others (60). Sunitinib is a multitargeted tyrosine kinase inhibitor that predominantly targets vascular endothelial growth factor (VEGF). In addition to its anti-VEGF activity, sunitinib has activity in suppressing T_REGs_, so it is an ideal compound to combine with MVA-5T4 to treat mRCC. Although this combination was studied in one of the three arms of the aforementioned phase III trial, given the problems of that study design and the strong scientific rationale, it is worth retesting this combination.

Finally, the rapid progression in targeted drug development represents a great opportunity to improve the clinical benefit of patients with cancer. Ipilimumab (anti-cytotoxic T-lymphocyte-associated antigen 4), anti-programed death 1, and anti-programed death ligand 1 represent a new family of targeted therapies that inhibit the inhibitory checkpoints of the immune system (61–63). The combination of MVA-5T4 and these novel immunomodulator drugs could be a good strategy to overcome cancer immune tolerance.

In conclusion, MVA-5T4 (TroVax^®^) is a novel vaccine therapy that has been proven safe and could potentially impact the treatment of various solid tumors, including RCC. Well-designed clinical trials based of both biomarkers and combinations with other and new immunomodulator agents are needed.

## Conflict of Interest Statement

The authors declare that the research was conducted in the absence of any commercial or financial relationships that could be construed as a potential conflict of interest.
